# New Online Manuscript Submission System of Balkan Medical Journal

**DOI:** 10.4274/balkanmedj.2019.1.001

**Published:** 2019-01-01

**Authors:** Zafer Koçak, Okan Çalıyurt

**Affiliations:** 1 Department of Radiation Oncology, Trakya University School of Medicine, Edirne, Turkey; 2 Department of Psychiatry, Trakya University School of Medicine, Edirne, Turkey

Immediately after I was appointed as the Editor-in-Chief in the fall of 2016, we had to face with a change in the publishing house. We successfully overcame this difficult process through the great efforts of our editorial team and Galenos publishing house. In 2017, we realized our goal of having a dynamic website that we dreamed of (1).

Balkan Medical Journal is one of the first journals to use the online submission system in Turkey. In 2006, we started using an online submission system for the first time (2). In 2013, we needed a better system because of the increase in the number of submissions, and hence, we started using the ScholarOne manuscript submission system.

Our performance with ScholarOne for the past 5.5 years is shown in Table 1. The rate of acceptance was below 10% in this 5.5 year period. The average time from submission to the first and final decision was approximately 14 and 16 days, respectively. Except from Turkey, we observed that the most number of published articles were from Balkan countries. Considering the number of article submissions outside Turkey, the researchers who submitted the most number of articles were from China, Iran, Serbia, and India.

However, in the second half of the year 2018, as a result of depreciation of Turkish lira, we had to reduce the costs of the journal. Being the editorial board, we started looking for an online submission system of comparable quality at a lower cost. After a long-term search by the editorial team and the publisher, we decided that the Manuscript Manager peer review system would meet all our needs. With this decision, starting with the last week of November 2018, we began using the new system. In this transition period, there may be some shortcomings and errors. Therefore, we hope that the authors and the reviewers will be tolerant.

The Manuscript Manager peer review system is a product of Akron ApS, based in Copenhagen, Denmark. They have more than 15 years of experience in working with peer review software for academic organizations. Some of the benefits of the new system include a user-friendly platform, no requirement of technical support or installation costs, and easy customization for your journal.

All processes related to the new article submission and review process should be made through the new online submission system available at https://www.manuscriptmanager.net/sLib/v4/login. php?paramScreen=FJ9enn8VfEwHq0NyAL1P9XnBjILxf94k4KO4DTwavsc=.

For detailed instructions on using Manuscript Manager, please visit the website at https://manuscriptmanager.com/site/. We hope that our authors, reviewers, and editors will adapt quickly to this new system.

## Figures and Tables

**Table 1:**
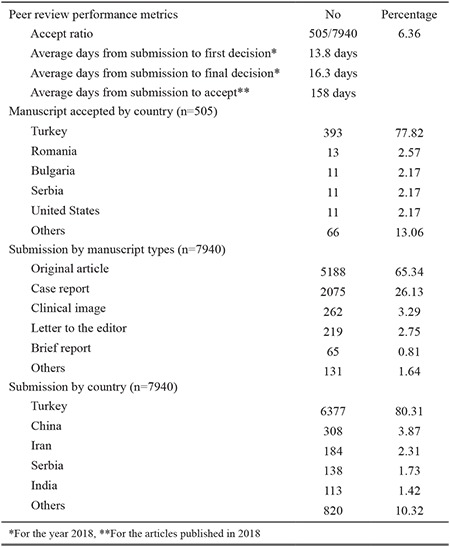
Peer review performance metrics and submission statistics for the past 5.5 years (ScholarOne period: June 2013 to December 2018)
